# Electrospun Polycrown Ether Composite Nanofibers as an Adsorbent for On-Line Solid Phase Extraction of Eight Bisphenols from Drinking Water Samples with Column-Switching Prior to High Performance Liquid Chromatography

**DOI:** 10.3390/polym14214765

**Published:** 2022-11-07

**Authors:** Tong Xu, Rui Zhang, Yueling Bi, Jingjing Li, Xiaohuan Li, Liqin Chen, Zhongze Fang

**Affiliations:** 1 Department of Toxicology and Sanitary Chemistry, School of Public Health, Tianjin Medical University, Tianjin 300070, China; 2 Department of Pharmacy, Tianjin Xiqing Hospital, Tianjin 300380, China; 3 Tianjin Key Laboratory of Environment, Nutrition and Public Health, Tianjin 300070, China

**Keywords:** bisphenols, polymeric crown ether composite nanofiber, on-line packed fiber solid phase extraction, HPLC-FLD

## Abstract

Bisphenols (BPs) are a class of endocrine disruptors widely existing in the environment. They have a great impact on human health owing to their environmental endocrine disrupting effects, chronic toxicity, neurotoxicity, cytotoxicity and genetic toxicity. In this paper, an on-line packed fiber solid phase extraction (PFSPE) coupling with column-switching HPLC-FLD determination method was developed for the determination of eight BPs in drinking water. The poly (dibenzo-18-crown-6-ether)/polystyrene composite nanofibers (PDB18C6/PS) were prepared by electrospinning and used as an adsorbent for the on-line PFSPE column. The on-line PFSPE-HPLC equipment contained a dual ternary pump and a switching valve to enable enrichment, purification, and analysis directly in the system. The results showed that the proposed on-line PFSPE-HPLC-FLD method realized the simultaneous separation and detection of eight BPs: BPF, BPE, BPA, BPB, BPAF, BPAP, BPC and BPZ. The curves of the target analytes were prepared with good correlation coefficient values (r^2^ > 0.998) in the range of 50–1000 pg/mL. The limit of detection (S/N = 3) was 20 pg/mL, the limit of quantitation (S/N = 10) is 50 pg/mL. The recoveries of eight BPs were 94.8–127.3%, and the intra-day precisions (RSD) were less than 10%. The PFSPE column made of the PDB18C6/PS composite nanofibers has stable properties and can be reused at least 200 times. In the detection of drinking water samples, BPZ was detected in nearly 80% of drinking water samples, and BPA, BPAP, BPF and BPAF were also detected in some water samples. This high level of integration and automation was achieved in pretreatment of eight BPs from water samples. The proposed simple, rapid, and practical method has been successfully applied to the detection of eight BPs in drinking water, which can provide powerful technical support for drinking water quality and safety monitoring.

## 1. Introduction

BPs (bisphenols, BPs) are a class of environmental endocrine disruptors (EDCs), which can be contacted and ingested by humans through air, water, soil, electronic equipment and food packaging materials [1]. EDCs have been known to interfere with endocrine systems by mimicking, blocking, and triggering actions of hormones and implicated with toxic effects, e.g., disorders in development and reproduction, for its molecular structure is very similar to that of hormones or some ligands in the human body [2,3]. Children’s exposure to BPA will lead to brain development impairment, attention deficit, and hyperactivity disorder (ADHD), and also lead to anxiety and metabolic-related diseases [4]. BPA is used to manufacture polycarbonate plastic and epoxy resins, and is widely used for a variety of applications, such as baby feeding bottles, food-can lining and sealants in dentistry; such an extensive use of BPA results in widespread human exposure in the general population [5]. With the continuous intake of accumulated food, it will have a cumulative effect in the human body and bring harm to human health. Due to the increasing restrictions on the use of BPA in food contact materials, many countries around the world have issued bans to prohibit the use of BPA in food contact materials and articles for infants and young children, some analogues of bisphenol have gradually become substitutes for BPA [6,7]. However, studies have shown that these substitutes still show environmental endocrine disrupting effects similar to BPA, as well as chronic toxicity, neurotoxicity, cytotoxicity, genotoxicity, and estrogen activity [8,9]. Therefore, the determination of BPs in environmental media and food contact material samples is of great significance to protect human health.

BPs are a kind of isomeric phenolic chemical formed by the aromatic condensation of two phenolic rings [10], mainly including BPF, BPE, BPA, BPB, BPAP, BPAF, BPC, BPZ, BPS and TBBPA, and are one of the frontier research hotspots in the field of environment in recent years. This kind of substance has a weak chemical polarity and complex sample matrix. The traditional BPs enrichment technology uses off-line liquid-liquid extraction and solid-phase extraction [11,12,13,14,15], which cannot realize the automatic on-line combination of sample pretreatment and detection. The off-line pretreatment method is not only time-consuming and laborious, but also affects the accuracy and precision of the whole analysis. In addition, the background contamination of bisphenol substances released from laboratory plastic products may also lead to significant errors, especially when detecting ultra-trace level of BPs [16]. Therefore, it has become a new research trend to explore an automatic on-line combination technology of sample pretreatment and detection [7,17,18,19]. The combination of on-line sample pretreatment and high-performance liquid chromatography can reduce the labor intensity of technicians, realize the high automation of the analysis process, and the solid phase extraction column can be recycled for several times, saving the analysis cost. Therefore, the possible pollution, pretreatment time, and consumption of organic solvent during sample transfer are reduced. More importantly, it can reduce or even eliminate the error caused by individual differences in manual operation and improve the sensitivity, accuracy and reproducibility of analysis. Up to now, there are few automatic on-line combinations of technology for sample pretreatment and detection available for simultaneously detecting a variety of BPs. Han has reported an online enrichment-HPLC-FLD method in simultaneous monitoring of bisphenols in children’s water bottles; however, manual syringe injection makes the entire analysis process not practically automated [17]. An on-line coupling of nanofibrous extraction with column-switching HPLC method has been established for the determination of bisphenol A in environmental water samples by Háková [20]. Only one BPA was detected with a LOQ of 2000 pg/mL, but this research also shows that nanofibers can be used as adsorbents for on-line SPE columns.

The technology of packed fiber solid phase extraction (PFSPE) based on electrospun nanofibers is developing rapidly, and its core is to use nanofibers to replace the current large particle size of micrometer solid phase adsorption materials for sample pretreatment. Depending on the physical and chemical properties of the captured molecules, nanofibers with selective interaction with captured molecules can be spun by high voltage electrospinning technology, which lays the foundation for the establishment of an on-line PFSPE and analysis platform for polar molecules [21,22,23]. In this experiment, an on-line PFSPE-HPLC-FLD automatic method was innovatively established based on the poly (dibenzo-18-crown-6-ether)/polystyrene composite nanofibers (PDB18C6/PS) used as SPE adsorbent. BPs in drinking water samples were enriched by nanofibers and directly entered the chromatographic system for separation and detection. This on-line enrichment, separation and detection method can simultaneously detect eight BPs, reducing the possible pollution and pretreatment steps in the process of sample transfer, and greatly improve the sensitivity and accuracy of analysis. Moreover, the high reuse efficiency of PFSPE column increases the convenience and cost-effectiveness of the method. In this study, the on-line pretreatment and detection of eight BPs can be realized at the same time with only an ordinary HPLC-FLD system and a switching valve, which not only provides an efficient on-line sample pretreatment and detection system for the measurement of trace BPs in drinking water, but also further expands the application range of on-line sample pretreatment technology using nanofibers as adsorbents.

## 2. Experimental

### 2.1. Materials

Bisphenol A (BPA), 4,4′-(1-Phenylethylidene) bisphenol (BPAP), 4,4′-Ethylidenebisphenol (BPE), 1,1-Bis (4-hydroxyphenyl) cyclohexane (BPZ), 4,4′-(Hexafluoroisopropylidene) diphenol (BPAF), and 4,4′-Dihydroxydiphenylmethane (BPF) were purchased from McLean Biochemical Technology CO., Ltd. (Shanghai, China). Bisphenol B (BPB) and 2,2-Bis (4-hydroxy-3-methylphenyl) propane (BPC) were purchased from Yien Chemical Technology CO., Ltd. (Shanghai, China). HPLC-grade methanol, acetonitrile and glacial acetic acid were obtained from Tianjin Chemical Reagent Company (Tianjin, China). All other reagents were of analytical grade unless otherwise indicated. Polystyrene (PS) (Mw = 185,000) was provided by Shanghai Chemical Agents Institute (Shanghai, China). Poly (dibenzo-18-crown-6-ether) (PDB18C6) was synthesized in the laboratory of Tianjin Medical University. Empty 10 × 2.1 mm and 10 × 4.6 mm column cartridge kits were purchased from Dalian Replete technology instrument CO., Ltd. (Dalian, China).

### 2.2. Instrumentation

HPLC with a fluorescence detector was carried out on an UltiMate3000 HPLC connected to an FLD-3100 fluorescence detector (Thermo Scientific, Waltham, MA, USA). The wavelengths of excitation and emission were 228 and 306 nm, respectively. A 2000 μL volume was injected into a YMC-Pack pro C_18_ column (100 × 3.0 mm, 5 μm particle size) via a WPS-3000 SL autosampler (loop volume of 2500 μL). An HPLC software package (Chromeleon 7.2 SR5) was used for the data analysis. The mobile phase for left pump was 5% methanol. The mobile phase of A for the right pump was ultrapure water. The mobile phase of B for the right pump was methanol. The flow rate was set at 0.5 mL/min and the temperature of the column oven was set to 35 °C.

### 2.3. Preparation of Standard Solutions and Samples

Stock solutions (1 mg/mL) of all analytes were prepared from their standards by dissolving them in methanol and stored in the dark at −20 °C. A mixture standard stock solution (0.1 mg/mL) was prepared in ultrapure water from each standard solution before use.

### 2.4. Preparation of Electrospun Nanofibers

The PDB18C6/PS composite nanofibers were fabricated by electrospinning as described elsewhere [24], and 10% PS and 5% PDB18C6 were mixed and electrospun to prepare a composite nanofiber for adsorbent in this study.

### 2.5. Preparation of Column for On-Line PFSPE and Off-Line PFSPE

The extraction columns for on-line PFSPE were prepared manually by packing the PDB18C6/PS composite nanofibers into an empty 10 × 2.1 mm (about 10 mg) or 10 × 4.6 mm (about 40 mg) column cartridge with two removable sieve plates [23]. The cartridge was placed in a guard column holder and connected to the system using HPLC fittings (as shown in Figure S1 in Supplementary Materials).

A 1 mL microcolumn was used as off-line PFSPE column. The PFSPE columns were prepared as described in a previous paper [22]. Filter support was not necessary for packing fiber (3 mg) into a 1 mL microcolumn. The off-line PFSPE column was firstly preconditioned by 100 μL of methanol and 100 μL of water before use.

### 2.6. The On-Line PFSPE-HPLC Analysis Procedure

The on-line PFSPE procedure was implemented by employing an on-line PFSPE column on the ten–port valve connected to an HPLC sampling loop located on a six-port valve. The ten–port valve was used to design a procedure for on-line pretreatment and analysis. The first step was on-line sample pretreatment; in this step, the sample was loaded by an autosampler into the on-line PFSPE column for extraction and purification. Secondly, elution and transfer of the target compounds to the analytical column were carried out by switching the ten–port valve (straight-flushing). The third step involved separation and determination of the eight BPs, and the ten–port valve was switched back at this time to equilibrate the PFSPE column for the next run. The total running time was 10 min. Figure S2 shows the schematic diagram of on-line sample pretreatment and transfer of the target compounds.

A 2000 μL aliquot of sample was injected into the on-line PFSPE column. When the BPs were preconcentrated on the PFSPE column, the analytical column was simultaneously equilibrated with the mobile phase. The valve switch time was set to be the same as the optimal extraction time at 3.5 min. Isocratic elution with 72% methanol/28% water lasted for 1 min to transfer the adsorbed targets to the analytical column. After that, the valve was switched back to balance the PFSPE column again and a gradient elution program for right pump was used for separation and determination of the eight BPs at the same time.

### 2.7. Preparation of Drinking Water

Four types of drinking water were collected from domestic supermarkets, domestic tap water, and filtered water, numbered from 1^#^ to 28^#^, including 19 brands of 27 different batches of plastic packaged drinking water (1^#^~19^#^) and barreled water (20^#^~22^#^), 6 different types of filtered water from 4 brands filters (23^#^~26^#^) and tap water from 2 companies (27^#^~28^#^). Of a total of 35 water samples, 5 mL water samples from each bottle were placed in test tubes for the determination of the concentration of BPs. Table S1 lists the characteristics of the water samples. Table S2 list the details and physical properties of the water samples collected in this study.

## 3. Results and Discussion

### 3.1. Optimization of Chromatographic Separation Conditions

The tested parameters of chromatographic separation conditions were the composition and the flow rate of the separation mobile phase and the analytical chromatographic column used.

**Figure 1:**
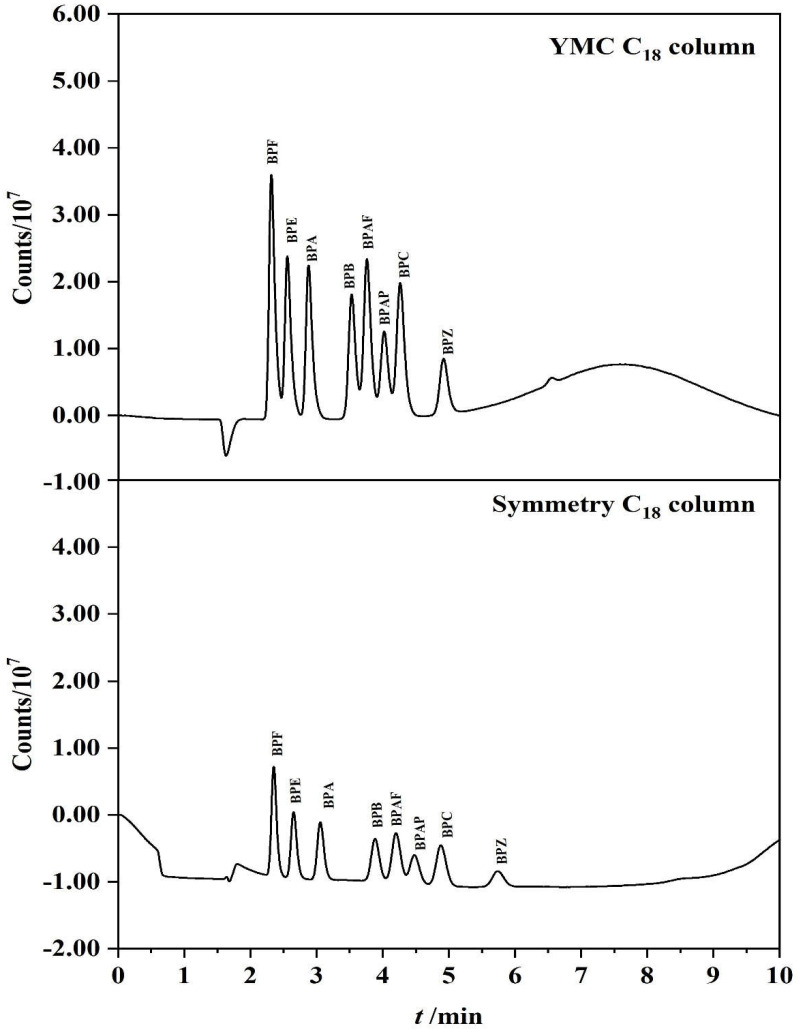
Comparison of separation and response of different chromatographic columns to the same BPs standard solution (YMC C_18_ column vs. Symmetry C_18_ column).

As for the chromatographic column and separation conditions used in the references, Symmetry C_18_ chromatographic column (4.6 × 150 mm, 5 μm) was firstly used as the separation chromatographic column, and the gradient ratio of the mobile phase was optimized at a flow rate of 0.9 mL/min. Six different procedures were carried out to compare the chromatography separation and signal response. Specific chromatograms and corresponding mobile phase conditions are shown in Figure S3. Finally, an optimal condition was obtained to separate the baseline of the eight BPs (Figure S3F). Under this optimal mobile phase condition, other chromatographic column was also tried for the experiment. The results show that the response value of YMC column is higher and the resolutions of the eight BPs are acceptable under this condition (as shown in Figure 1). Therefore, by comparing the proportion of mobile phase and the chromatographic column, two experimental conditions for the establishment of on-line method were finally determined. The YMC-Pack pro C_18_ column (100 × 3.0 mm, 5 μm) was selected as the chromatographic column for subsequent experiments. A gradient procedure (as shown in Figure S3F) was selected for following on-line separation of the eight BPs. The corresponding flow rate was changed to 0.5 mL/min, because the inner diameter of the YMC column is relatively small.

### 3.2. The Influence of the Desorption Solvent

**Figure 2:**
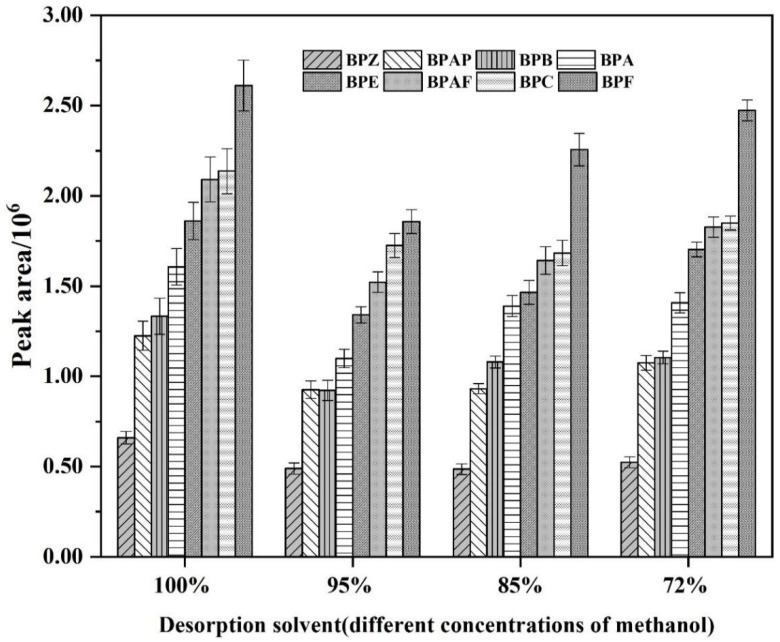
The response values of eight BPs under different concentrations of desorption solvent (from 100% to 72% methanol).

Desorption solvent with different concentrations have different elution capacities, which directly affects the concentration of the analytes to be detected entering the fluorescence detector. Desorption solvent should effectively retain the target compounds, and meanwhile not to affect the subsequent on-line separation and detection. A total of four concentrations of desorption solvent were tested in this experiment, namely 100% methanol, 95% methanol, 85% methanol, and 72% methanol. First, 500 μL of standard solution (100 ng/mL) was loaded onto an off-line PFSPE column and pushed through the column, then it was washed with 100 μL of water, then 100 μL of desorption solvent was loaded onto the PFSPE column and collected to verify the elution ability. The injection volume was 10 μL. The results show that the peak area of each analyte eluted with 100% methanol is the largest, followed by 72% methanol, 95% methanol, and 85% methanol. The peak area change with methanol concentration is shown in Figure 2. However, in the on-line experiment, it was found that the use of 100% methanol as desorption solvent would lead to the subsequent baseline instability, and the separation of analytes was greatly affected. Therefore, the use of 72% methanol as desorption solvent not only has strong elution ability, but also has a constant proportion with the initial mobile phase, which has no influence on the subsequent separation and detection. Considering comprehensively, 72% methanol was selected for desorption solvent.

### 3.3. Optimization of the On-Line PFSPE Procedure

The design of on-line PFSPE procedure is the core of whether the target analytes can be well enriched, purified and completely separated. It is mainly through setting the time program, controlling the switch of valve position and the gradient of mobile phase, to achieve the purpose of automatic on-line pretreatment and analysis of sample. In the separation optimization experiments, key experimental conditions such as desorption solvent, mobile phase gradient, column, and flow rate have been determined. On this basis, only the enrichment time and the carrier mobile phase of the left pump need consideration to establish the on-line PFSPE procedure.

**Figure 3:**
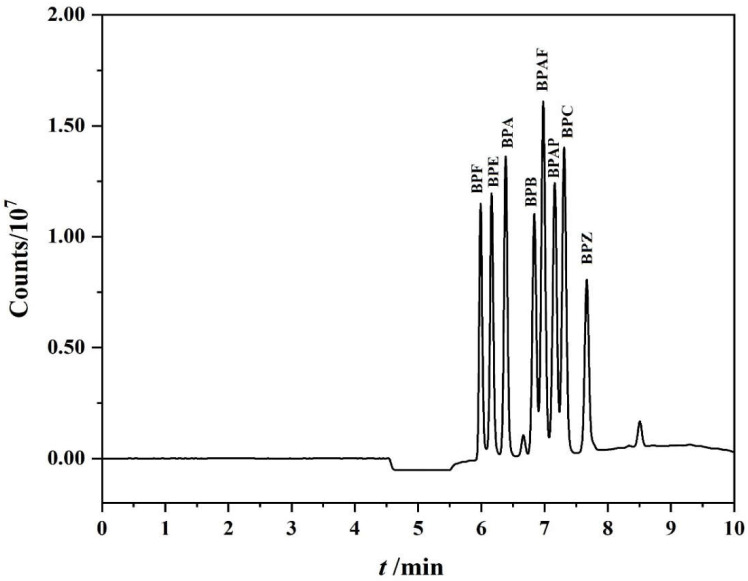
The chromatogram of on−line enrichment and separation of the eight BPs under the optimization procedures.

The carrier mobile phase of the left pump is required not only to transport the sample to the PFSPE column, but preferably to remove some matrix impurities at the same time. The mixtures of the methanol and water were tested at concentrations ranging from 0 to 20%. Figure S4 shows that the response of target substance was the largest when 5% methanol was used as carrier mobile phase. Therefore, 5% methanol in water was chosen for this carrier mobile phase. Five kinds of enrichment time were tested from 2.5 min to 5.5 min. As shown in Figure S5, the best response value was obtained under the duration at 3.5 min; therefore, the valve switching time was set at 3.5 min. The right pump offers sample elution and sample separation, a gradient of ultrapure water (mobile phase A) and methanol (mobile phase B) was used as the sample desorption solvent and the separation mobile phase. Therefore, this experiment tested the chromatographic separation and signal response under different procedures to screen out the best separation and enrichment conditions. The optimal procedure for on-line separation and enrichment of eight BPs is shown in Table 1 and the chromatogram is shown in Figure 3. Under this condition, the whole running time takes 10 min, the eight BPs are well separated and the response value is high.

### 3.4. Selecting PFSPE Column Types and Injection Volume

The PDB18C6/PS material exhibits stable behavior and has strong adsorption capacity for polar substances; in this experiment, the BPs solution was tested after passing through the off-line PFSPE column, and it was found that there was no peak response of BPs in the solution, indicating that the PDB18C6/PS composite nanofiber material had a high adsorption performance for BPs.

**Figure 4:**
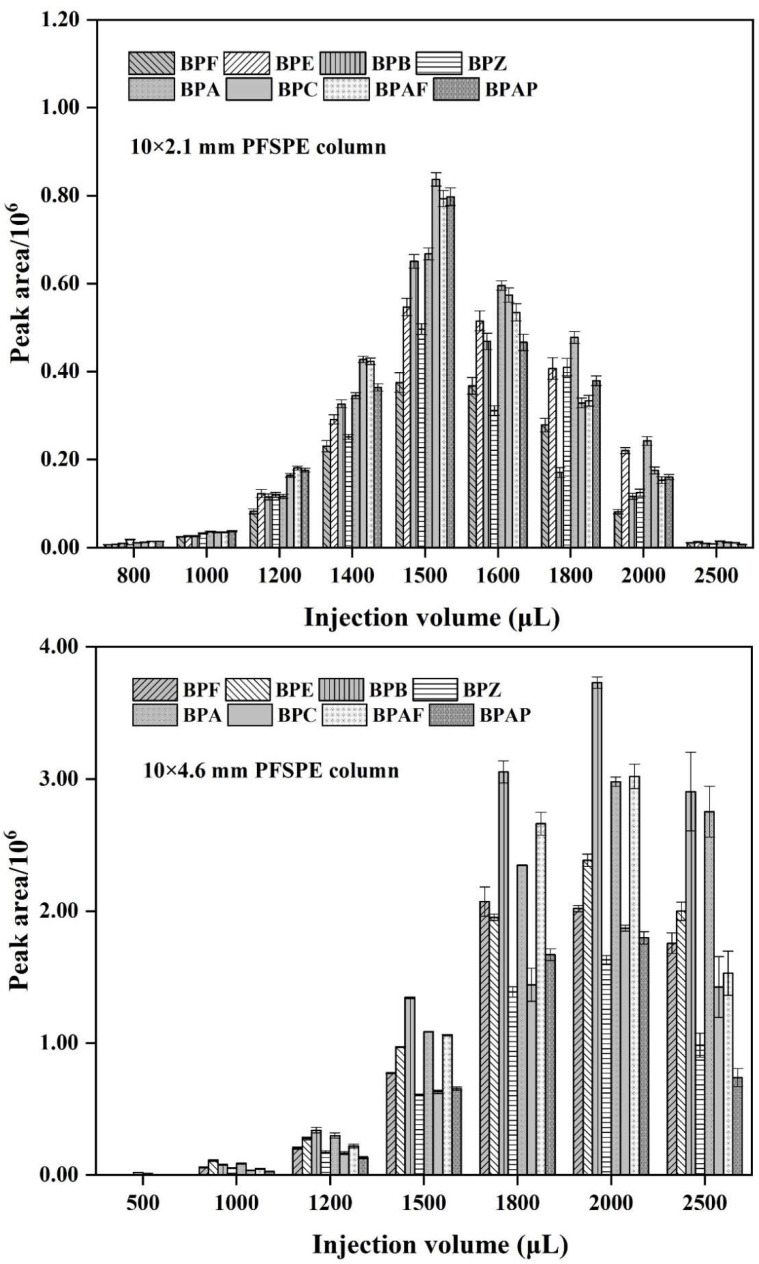
Comparison of response values of eight BPs under different injection volumes (10 × 2.1 mm PFSPE column vs. 10 × 4.6 mm PFSPE column).

In the selection of column type, two types, 10 × 2.1 mm and 10 × 4.6 mm, were compared. Different injection volumes from 500 to 2500 μL were taken to obtain the column chart of their response values under different injection volumes (as shown in Figure 4). For the injection detection, 1 ng/mL BPs mixed standard solution was used, and each volume was injected three times. The difference of the injection volume affects the adsorption efficiency of BPs on the PFSPE column. It was found that the peak area of each target analyte showed a trend of increasing significantly and then decreasing with the increase of injection volume. The trend of the two types of PFSPE columns is basically the same. Since the fiber packing amount of 10 × 4.6 mm PFSPE column is about 4 times that of 10 × 2.1 mm PFSPE column, and the maximum response between the two is also 4 times different, it can be seen that the packing amount of the nanofibers also affect the adsorption amount of PFSPE column to the target analytes. The peak area was reached maximum when the injection volume was 1500 μL for 10 × 2.1 mm PFSPE column and 2000 μL for 10 × 4.6 mm PFSPE column, respectively. Different types of PFSPE columns have different injection volumes to achieve the best adsorption. From the analysis of experimental results, the adsorption capacity of 10 × 4.6 mm PFSPE column is much larger than that of 10 × 2.1 mm PFSPE column, and the injection volume can be selected as 2000 μL. Therefore, 10 × 4.6 mm PFSPE column and 2000 μL of injection volume were determined in this experiment.

### 3.5. Stability of Nanofibers during On-Line PFSPE Chromatography Testing

In previous experiments, it was found that after hundreds of uses of PFSPE column, the nanofibers had only slight mechanical deformation, no significant change was observed in the diameter and the nanostructure remained unchanged [23]. In this experiment, the repeated use times of the PFSPE column were counted to further investigate the stability of the PFSPE column, and a total of 211 tests were performed. The Figure S6 shows that the peak area of each substance obtained in the 211 tests do not change significantly, indicating that the PFSPE column made of the composite nanofibers has strong stability and is suitable for use in on-line PFSPE-HPLC system.

### 3.6. Stability Test of BPs

Samples of 5 ng/mL standard solution spiked in water were stored at room temperature. The samples were injected and analyzed at the same time point for three consecutive days, the first day, 24 h later and 48 h later, the injection volume is 1500 μL. Record the peak area of each BPs and then calculate the degradation rate at 24 h and 48 h, respectively. The calculation results shown in Figure S7 indicated that the eight BPs degraded rapidly within 24 h, and slowly degraded between 24 h to 48 h. At 48 h, BPZ had the highest degradation rate, reaching 23.7%. The lowest degradation rate at 48 h was obtained from BPA, with a degradation rate of 6.2%. From the stability test data of BPs at room temperature, it can be known that BPs are in poor stability at room temperature, which suggests that the relevant detection experiments should be completed at room temperature as soon as possible to avoid experimental errors caused by poor sample stability.

### 3.7. Linearity and Recovery of the On-Line PFSPE-HPLC Method

Validation of the on-line PFSPE-HPLC method using PDB18C6/PS composite nanofibers as the adsorbent was carried out to demonstrate the suitability of the method for determining BPs in water samples. The validation parameters are summarized in Table 2.

Method linearity was tested in the range from 10 to 1000 pg/mL using seven points. Taking the BPs concentration as the abscissa and the corresponding average peak area at each concentration as the ordinate, the standard curves of each BPs were drawn respectively. Standard solution containing BPs were prepared in ultrapure water at various concentrations (10, 20, 50, 100, 200, 500, 1000 pg/mL), 2000 μL of the mixture was injected into the HPLC system for further on-line pretreatment and analysis. There were good linear relationships in the range of 50~1000 pg/mL for all BPs. The linear correlation coefficients were all more than 0.998, the detection limit (S/N = 3) was 20 pg/mL, the limit of quantitation (S/N = 10) was 50 pg/mL.

The accuracy parameter was evaluated by the determination of the recovery using a standard addition procedure with water samples spiked at three concentration levels (50, 500, 1000 pg/mL), each in triplicate. The obtained recovery values are also shown in Table 2. The results showed that the standard recovery of the eight BPs was in the range of 94.8~127.3%, the relative standard deviation (RSD) was less than 10%; © recovery and precision meet the test requirements, indicating that the detection method established in this experiment is suitable for the quantitative analysis of BPs in the water sample.

### 3.8. Analysis of Real Samples

The optimized analytical method has been used to check the presence of BPs in 35 commercial water samples. The concentrations detected in each water sample of BPs are shown in Table 3. The results showed that 80% of the investigated drinking water samples were contaminated with BPZ, and only a few barreled water samples showed BPA, indicating that BPZ began to appear as a BPA substitute in the bottled containers of drinking water. It is also important to note that several emerging BPs emerged in this determination, such as BPF, BPAF and BPAP.

**Figure 5:**
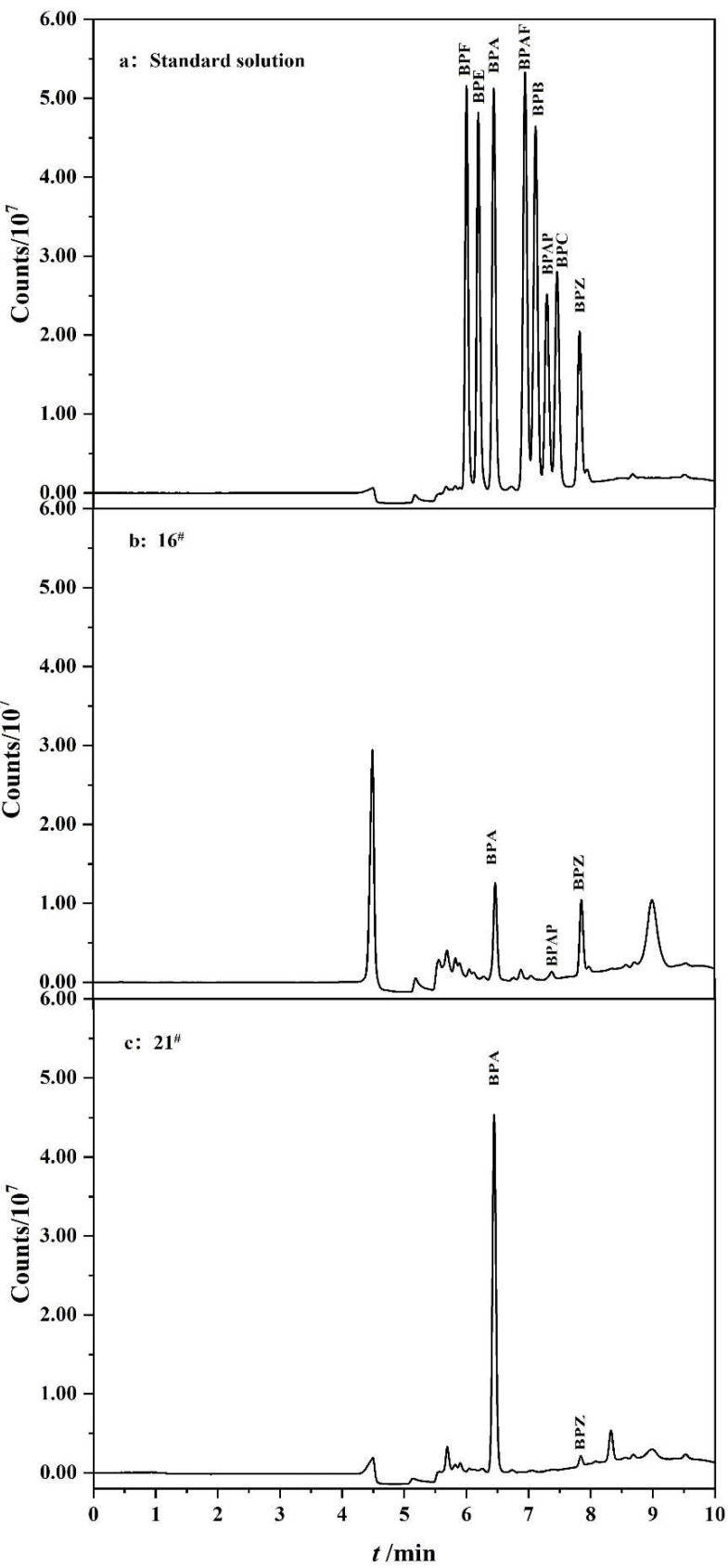
Chromatograms of (**a**) standard solution of eight BPs; (**b**) the extract of 16^#^ plastic−packaged drinking water; (**c**) the extract of 21^#^ barreled water.

The chromatograms of two water samples (16^#^ and 21^#^) are shown in Figure 5. After on-line enrichment and purification by PFSPE column, BPs could be well separated and detected in the HPLC-FLD system with excellent peak shape and good separation with impurities, which is convenient for the qualitative and quantitative analysis of BPs. In this method, the time required for on-line enrichment, purification and detection of the sample is within 10 min, which is convenient and quick, and greatly saves manpower and material resources.

### 3.9. Comparison of Analytical Performance with Available Methods

A comparison of the analytical performance of the developed method with reported on-line methods [17,18,19,20,25,26] is summarized in Table 4. To the best of our knowledge, in addition to this study, there is one study which reported on-line pretreatment coupled with HPLC to determine multiple BPs in water matrix [17]. The remaining studies reported only one or two types of BPs. As can be seen in Table 4, only HPLC-FLD can also provide relatively low LOD values, except for the low LOD of mass spectrometry method. In addition, compared with the other two on-line methods that also use nanofibers as adsorbents, our study allows for larger sample processing volumes (2 mL vs. 50 µL) and thus also provides relatively lower LOD values (20 pg/mL vs. 600 pg/mL). Furthermore, the entire running time of this method is 10 min, which is also advantageous among all methods in determination of multiple BPs.

## 4. Conclusions

In this study, an on-line PFSPE-HPLC-FLD automatic pretreatment and determination method was innovatively established which could simultaneously detect eight BPs in drinking water samples within 10 min. Under this method, the curves of the target analytes were prepared with good correlation coefficient values (r^2^ > 0.998) in the range of 20~1000 pg/mL. In addition, the PDB18C6/PS composite nanofiber as adsorbent for solid phase extraction of BPs was again confirmed to have strong stability. An on-line PFSPE column packing with this composite nanofiber can be reused for more than 200 times, which can reduce the cost of sample pretreatment and ensure the stability and repeatability of test data.

Judging from the test results of drinking water of different brands in this experiment, although BPA was detected in only four drinking water samples, BPZ is detected in nearly 80% of drinking water samples, indicating that food safety monitoring still has a long way to go, and the use of emerging BPs must be paid more attention to. This developed on-line PFSPE-HPLC-FLD method will provide strong technical support for food safety monitoring.

## Figures and Tables

**Table 1:** The optimization procedure for on-line separation and enrichment of eight BPs.

Time (min)	Left Pump	Right Pump		Valve Position
	A (%)	A (%)	B (%)	
0 3.5 4.5 4.55 5.6 7.7 7.8 10	100	28	72	10_1
	100	28	72	1_2
	100	28	72	10_1
	100	28	72	10_1
	100	20	80	10_1
	100	20	80	10_1
	100	28	72	10_1
	100	28	72	10_1

Flow rate: 0.5 mL/min.

**Table 2:** The linearity, repeatability, recoveries, LODs and LOQs for eight BPs.

	BPF	BPE	BPA	BPB	BPAF	BPAP	BPC	BPZ
Calibration parameters								
Slope	2913.4	2789.4	3347.8	3990	3379.2	1683.7	2004.4	1424.9 19898 0.9982 50.0–1000.0
Intercept	−61,320	108,056	−5056.9	−48,949	−36,578	−20,958	−22,048	
r^2^	0.9982	0.9999	0.9994	0.9988	0.9993	0.9987	0.9991	
Linear range (pg/mL)	50.0–1000.0	50.0–1000.0	50.0–1000.0	50.0–1000.0	50.0–1000.0	50.0–1000.0	50.0–1000.0	
Precision of the analytical method								
Intra-day repeatability (RSD %, *n* = 6)								
Spiking level (pg/mL)								
50.0	1.8	6.9	2.9	8.9	6.9	4.1	5.7	7.2 1.5 7.4
500.0	1.1	3.0	2.3	0.5	0.9	1.9	2.0	
1000.0	4.1	0.5	0.9	0.8	1.6	3.1	6.3	
Accuracy of the analytical method (Recovery, %)								
Spiking level (pg/mL)								
50.0	127.3	120.4	110.6	100.0	105.0	110.6	118.2	126.2 98.2 102.7 20 50
500.0	94.8	96.2	98.0	100.1	99.1	98.1	96.6	
1000.0	101.2	100.9	100.5	100.0	100.3	100.5	100.8	
LOD (pg/mL)	20	20	20	20	20	20	20	
LOQ (pg/mL)	50	50	50	50	50	50	50	

RSD: relative standard deviation; *n*: number of determinations.

**Table 3:** Contamination levels of BPs in drinking water samples from local markets (pg/mL).

Sample No.	BPZ	BPF	BPA	BPAF	BPAP
01	164.5	-	-	-	-
02-1	+	-	-	-	-
02-2	72.8	-	-	-	-
02-3	203.2	-	-	-	-
04-1	666.2	-	-	-	-
04-2	195.6	-	-	-	-
05	+	-	-	-	-
06	95.7	-	-	-	-
07	128.6	-	-	-	-
08-1	1131.5	-	-	-	-
08-2	486.6	-	-	-	-
09	49.7	-	-	-	-
10	49.7	-	-	-	-
11	192.2	-	-	-	-
12	335.1	-	-	-	-
13	85.8	-	-	-	-
14	733.9	-	-	-	-
15	115.3	1600.0	-	847.7	-
16	441.0	-	257.7	-	56.2
17	254.9	-	-	-	-
18	268.0	-	-	-	-
20	899.1	-	2143.5	-	612.2
21	+	-	915.1	-	-
22	220.2	-	694.4	-	-
26-1	340.1	-	-	-	-
26-2	+	-	-	-	-
28	57.2	-	-	-	-

+ Represents the content is under LO–. - Represents the content is not detected.

**Table 4:** Comparison of analytical performance of the proposed method with existing on-line methods for detection of BPs in water samples.

Sample Preparation	Analytes	Linearity	LOD	Colum Size	Sample Volume	Run Time (min)	Detector	Ref. & Year
On-line SPE poly (EDMA-GMA) monoliths	BPA	1.0–160 (pg/mL)	0.3 (pg/mL)	50×4.6 mm	100 mL	16	LC–ESI /MS/MS	[25] 2008
On-line column-switching SPE (Polyamide 6 nanofiber)	BPA	2–500 (ng/mL)	0.6 (ng/mL)	5 × 4.6 mm	50 µL	5	FD 225/320 nm	[20] 2018
On-line SPE (Hypersil Gold aQ C18)	BPA	0.80–2.8 (pg/mL)	0.5 (pg/mL)	20 × 2 mm	10 mL	15.5	UHPLC–MS/MS	[19] 2019
SIA On-line SPE (SDB-RPS)	BPA 4tBP	5–80 (ng/mL)	0.77 (ng/mL) 1.46 (ng/mL)	10 × 10 mm	15 mL	82 8	HPLC–UV 225 nm	[18] 2020
On-line SPE (C18)	BPA BPB BPC BPP BPZ BHPF BPAF BPAP TMBPA	0.4–80.0 (pg/mL) (BPA, BPB)	0.13 (pg/mL) (BPA, BPB)	150 × 4.6 mm	5 mL	30	LC–FD 228/306 nm 230/319 nm	[17] 2021
		0.2–80.0 (ng/mL) (BPHF)	66.7 (pg/mL) (BPHF)					
		4.0–800.0 (pg/mL) (BPAP, TMBPA)	1.33 (pg/mL) (BPAP, TMBPA)					
		10.0–800.0 (pg/mL) (BPAF, BPC, BPP)	0.67 (pg/mL) (BPZ)					
		2.0–800.0 (pg/mL) (BPZ)	3.33 (pg/mL) others					
On-line SPE (polyamide 6 nanofibrous)	BPA BPS BTP FXC	0.1–50 (μg/mL)	30 ng/mL	5 × 4.6 mm	50 µL	5	UHPLC–UV 230 nm	[26] 2022
		0.1–50 (μg/mL)	30 ng/mL					
		0.2–50 (μg/mL)	60 ng/mL					
		0.1–50 (μg/mL)	30 ng/mL					
On-line PFSPE (PS/PDB18C6 composite nanofibers)	BPA BPB BPC BPE BPF BPZ BPAF BPAP	50–1000 (pg/mL)	20 (pg/mL)	10 × 4.6 mm	2 mL	10	LC–FD 228/306 nm	This work

## Data Availability

The data presented in this study is available in Supplementary Material.

## Supplementary Materials

Supplementary data related to this article can be found at https://www.mdpi.com/article/10.3390/polym14214765/s1. Figure S1. The preparation of the on-line PFSPE column and connection to the HPLC system. Figure S2. The schematic diagram of on-line sample pretreatment and transfer of the target compounds. Figure S3. BPs separation under different gradient programs. Figure S4. Comparison of peak area of the BPs at different carrier mobile phase. Figure S5. Comparison of peak area of the BPs at different durations. Figure S6. Change of peak area of BPs with the number of PFSPE columns used (10 × 2.1 mm PFSPE column, 5 ng/mL standard solution spiked in water). Figure S7. Degradation rate of 8 BPs at 24 h and 48 h (10 × 2.1 mm PFSPE column, 5 ng/mL standard solution spiked in water). Table S1. The characteristics of drinking water samples. Table S2. The details and physical properties of drinking water samples.

## References

[1] Saini S.S., Martini M.F. (2021). A green hybrid microextraction for sensitive determination of bisphenol A in aqueous samples using three different sorbents: Analytical and computational studies. Microchem. J..

[2] Amir S., Shah S.T.A., Mamoulakis C., Docea A.O., Kalantzi O.I., Zachariou A., Calina D., Carvalho F., Sofikitis N., Makrigiannakis A. (2021). Endocrine Disruptors Acting on Estrogen and Androgen Pathways Cause Reproductive Disorders through Multiple Mechanisms: A Review. Int. J. Environ. Res. Public Health.

[3] Scsukova S., Rollerova E., Bujnakova Mlynarcikova A. (2016). Impact of endocrine disrupting chemicals on onset and development of female reproductive disorders and hormone-related cancer. Reprod. Biol..

[4] Hansen J.B., Bilenberg N., Timmermann C.A.G., Jensen R.C., Frederiksen H., Andersson A.M., Kyhl H.B., Jensen T.K. (2021). Prenatal exposure to bisphenol A and autistic- and ADHD-related symptoms in children aged 2 and5 years from the Odense Child Cohort. Environ. Health.

[5] Geens T., Goeyens L., Covaci A. (2011). Are potential sources for human exposure to bisphenol-A overlooked?. Int. J. Hyg. Environ. Health.

[6] Santonicola S., Albrizio S., Ferrante M.C., Raffaelina M. (2021). Study on bisphenol F, a bisphenol A analogue, at a dairy company: Health hazard and risk assessment. Food Chem. Toxicol..

[7] Jo M.J., Park J.H., An K.A., Choi H., Kang Y.S., Hwang M. (2020). Quantification of bisphenols in Korean urine using online solid-phase extraction-high-performance liquid chromatography-tandem mass spectrometry. Environ. Toxicol. Pharmacol..

[8] Ho S.M., Rao R., To S., Schoch E., Tarapore P. (2017). Bisphenol A and its analogues disrupt centrosome cycle and microtubule dynamics in prostate cancer. Endocr. Relat. Cancer.

[9] Beg M.A., Sheikh I.A. (2020). Endocrine disruption: Molecular interactions of environmental bisphenol contaminants with thyroid hormone receptor and thyroxine-binding globulin. Toxicol. Ind. Health.

[10] Rochester J.R. (2013). Bisphenol A and human health: A review of the literature. Reprod. Toxicol..

[11] Russo G., Barbato F., Grumetto L. (2016). Development and Validation of a LC-FD Method for the Simultaneous Determination of Eight Bisphenols in Soft Drinks. Food Anal. Methods.

[12] Zhang J., Zang L., Wang T., Wang X., Jia M., Zhang D., Zhang H. (2020). A solid-phase extraction method for estrogenic disrupting compounds based on the estrogen response element. Food Chem..

[13] Maragou N.C., Thomaidis N.S., Theodoridis G.A., Lampi E.N., Koupparis M.A. (2020). Determination of bisphenol A in canned food by microwave assisted extraction, molecularly imprinted polymer-solid phase extraction and liquid chromatography-mass spectrometry. J. Chromatogr. B Anal. Technol. Biomed. Life Sci..

[14] Guo X., Huang Y., Yu W., Yu X., Han X., Zhai H. (2020). Multi-walled carbon nanotubes modified with iron oxide and manganese dioxide (MWCNTs-Fe_3_O_4_−MnO_2_) as a novel adsorbent for the determination of BPA. Microchem. J..

[15] Alnaimat A.S., Barciela-Alonso M.C., Bermejo-Barrera P. (2019). Determination of bisphenol A in tea samples by solid phase extraction and liquid chromatography coupled to mass spectrometry. Microchem. J..

[16] Yoshiyuki W., Takuya K., Hiroe I., Masatoshi M., Nobuo T., Hosoy K. (2004). Reducing Bisphenol A contamination from analytical procedures to determine ultralow levels in environmental samples using automated HPLC microanalysis. Anal. Chem..

[17] Han S., Song Y., Hu J., Liu R., Chi Y., Kang A., Deng H., Zhu D. (2021). Novel computer-assisted separation prediction strategy for online-enrichment-HPLC-FLD in simultaneous monitoring of bisphenols in children’s water bottles. Food Chem..

[18] Villarreal-Morales R., Hinojosa-Reyes L., Hernández-Ramírez A., Ruíz-Ruíz E., Maya Treviño M.d.L., Guzmán-Mar J.L. (2020). Automated SPE-HPLC-UV methodology for the on-line determination of plasticisers in wastewater samples. Int. J. Environ. Anal. Chem..

[19] Goeury K., Vo Duy S., Munoz G., Prevost M., Sauve S. (2019). Analysis of Environmental Protection Agency priority endocrine disruptor hormones and bisphenol A in tap, surface and wastewater by online concentration liquid chromatography tandem mass spectrometry. J. Chromatogr. A.

[20] Háková M., Havlíková L.C., Chvojka J., Solich P., Satinsky D. (2018). An on-line coupling of nanofibrous extraction with column-switching high performance liquid chromatography—A case study on the determination of bisphenol A in environmental water samples. Talanta.

[21] Kang X., Pan C., Xu Q., Yao Y., Wang Y., Qi D., Gu Z. (2007). The investigation of electrospun polymer nanofibers as a solid-phase extraction sorbent for the determination of trazodone in human plasma. Anal. Chim. Acta.

[22] Chen L.Q., Wang H., Xu Z., Zhang Q.Y., Liu J., Shen J., Zhang W.Q. (2018). High-throughput and selective solid-phase extraction of urinary catecholamines by crown ether-modified resin composite fiber. J. Chromatogr. A.

[23] Chen L.Q., Tang Y., Xu B., Xu Z., Shen J., Zhang W.Q. (2020). Automated on-line packed fiber solid phase extraction for determination of urinary catecholamines. J. Chromatogr. B Anal. Technol. Biomed. Life Sci..

[24] Chen L.Q., Zhu X.H., Huang D.N., Xu Z., Shen J., Zhang W.Q. (2017). Polystyrene/poly(dibenzo-18-crown-6) composite nanofibers for the selective adsorption of plasma catecholamines. RSC Adv..

[25] Li L., Wang J., Zhou S., Zhao M. (2008). Development and characterization of an immunoaffinity monolith for selective on-line extraction of bisphenol A from environmental water samples. Anal. Chim. Acta.

[26] Erben J., Klicova M., Klapstova A., Hakova M., Lhotská I., Zatrochová S., Šatínský D., Chvojka J. (2022). New polyamide 6 nanofibrous sorbents produced via alternating current electrospinning for the on-line solid phase extraction of small molecules in chromatography systems. Microchem. J..

