# Supplementary material for: Electrospun Polycrown Ether Composite Nanofibers as an Adsorbent for On-Line Solid Phase Extraction of Eight Bisphenols from Drinking Water Samples with Column-Switching Prior to High Performance Liquid Chromatography
Source: Polymers (Basel). 2022 Nov 7;14(21):4765. doi: 10.3390/polym14214765 (PMC9659129; doi:10.3390/polym14214765)
Supplement: Supplementary file 1 [file polymers-14-04765-s001.zip › polymers-1958981-supplementary.pdf]

Electrospun Polycrown Ether Composite Nanofibers as an Adsorbent for On-Line Solid Phase Extraction of Eight Bisphenols from Drinking Water Samples with Column-Switching Prior to High Performance Liquid Chromatography

Tong Xu [1], Rui Zhang [1], Yueling Bi [2], Jingjing Li [1], Xiaohuan Li [1], Liqin Chen [1,3,*] and Zhongze Fang [1,3,*]

[1] Department of Toxicology and Sanitary Chemistry, School of Public Health, Tianjin Medical University, Tianjin 300070, China
[2] Department of Pharmacy, Tianjin Xiqing Hospital, Tianjin 300380, China
[3] Tianjin Key Laboratory of Environment, Nutrition and Public Health, Tianjin 300070, China
* Correspondence: chenliqin@tmu.edu.cn (L.C.); fangzhongze@tmu.edu.cn (Z.F.)

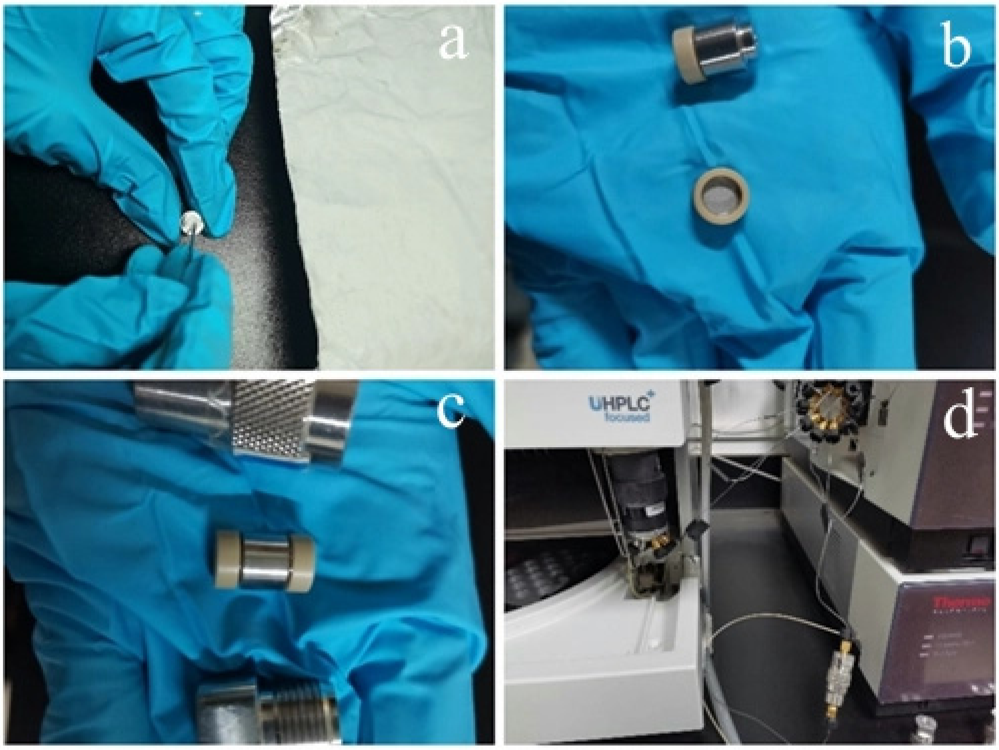

Figure S1. The preparation of the on-line PFSPE column and connection to the HPLC system. a: nanofiber packed into empty column cartridge; b: prepared PFSPE column; c: prepared PFSPE column with guard column holder; d: PFSPE column connected to HPLC system

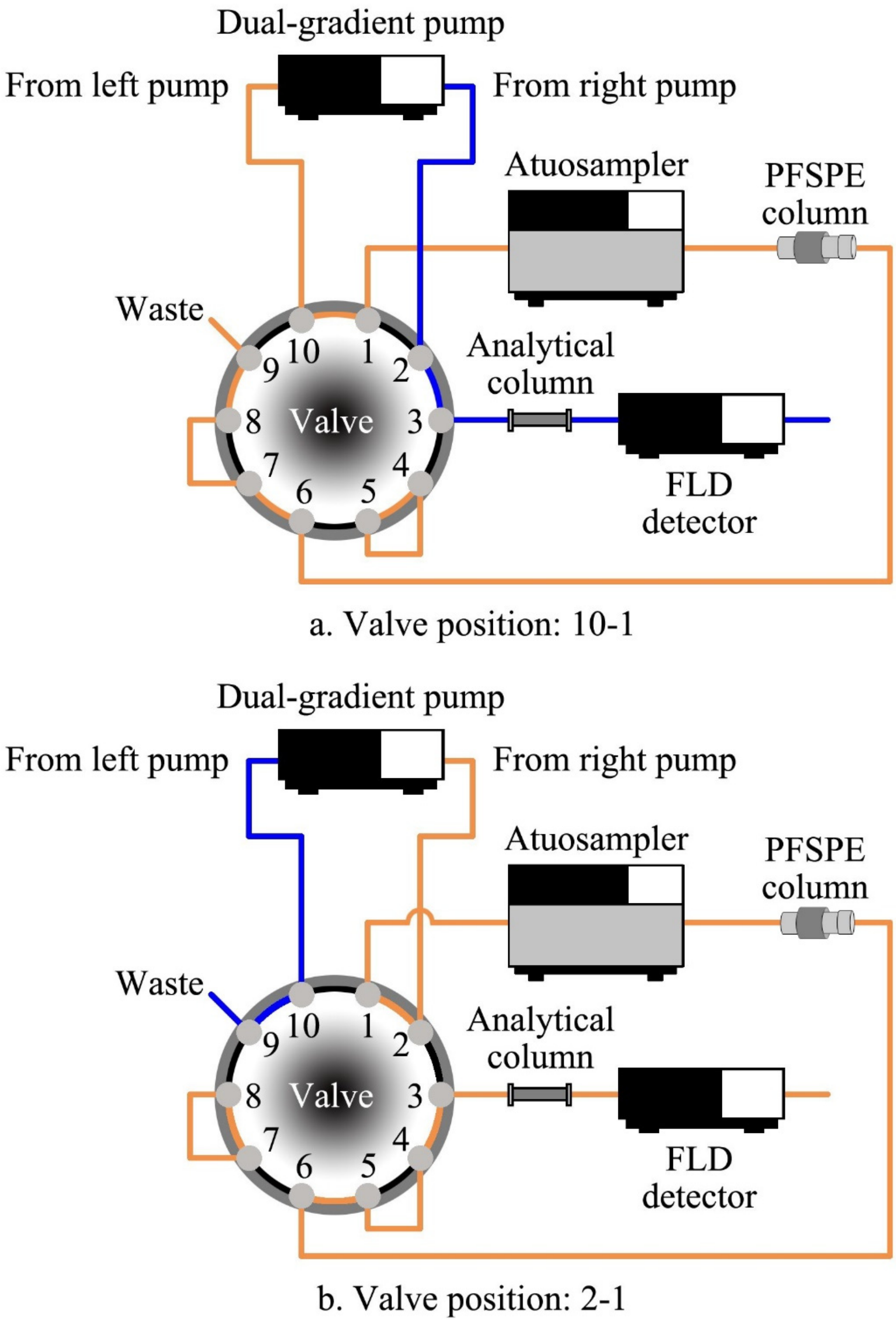

Figure S2. The schematic diagram of on-line sample pretreatment and transfer of the target compounds

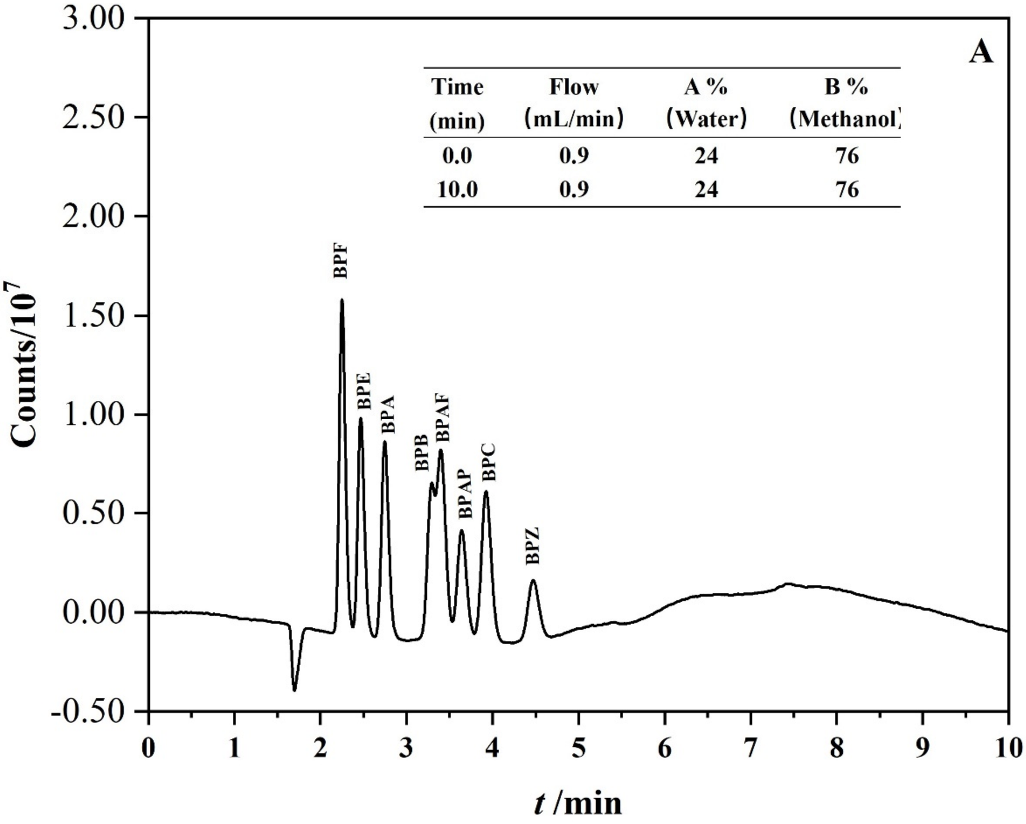

Time (min)	Flow (mL/min)	A % (Water)	B % (Methanol)
0.0	0.9	24	76
10.0	0.9	24	76

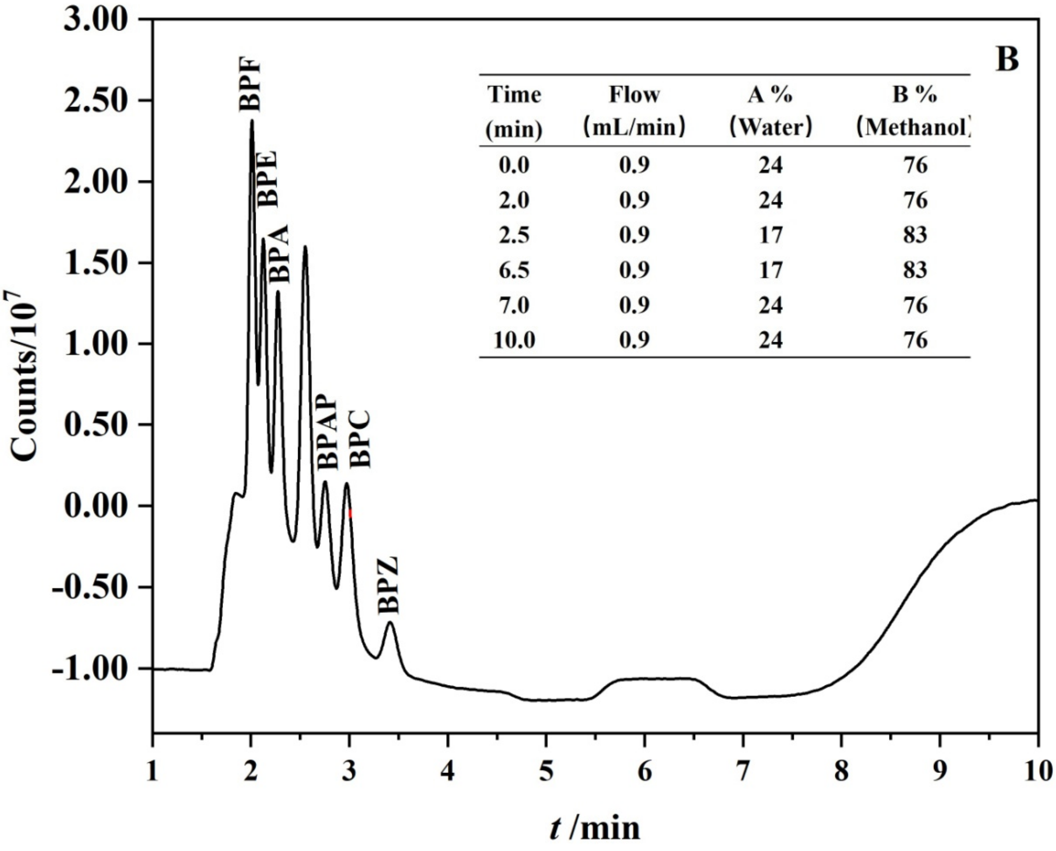

Time (min)	Flow (mL/min)	A % (Water)	B % (Methanol)
0.0	0.9	24	76
2.0	0.9	24	76
2.5	0.9	17	83
6.5	0.9	17	83
7.0	0.9	24	76
10.0	0.9	24	76

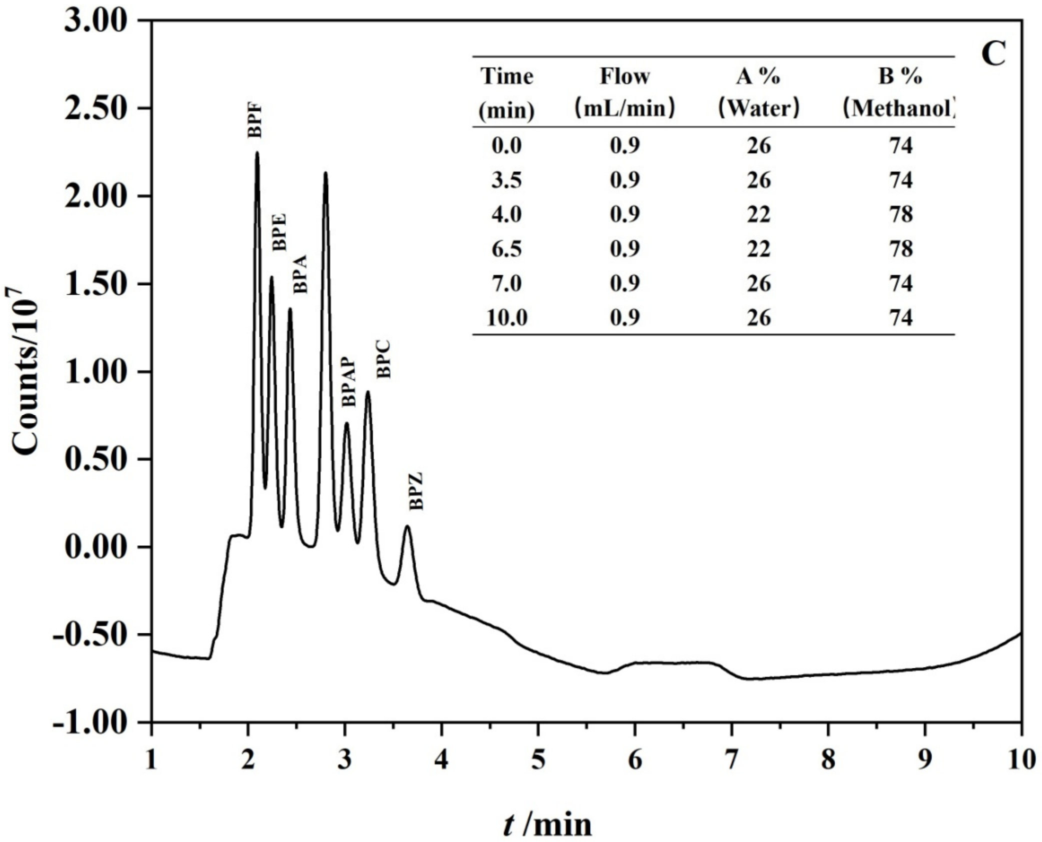
C
Time (min)
Flow (mL/min)
A % (Water)
B % (Methanol
0.0 0.9 26 74
3.5 0.9 26 74
4.0 0.9 22 78
6.5 0.9 22 78
7.0 0.9 26 74
10.0 0.9 26 74
BPF
BPE
BPA
BPAP
BPC
BPZ
Counts/10^7
t /min

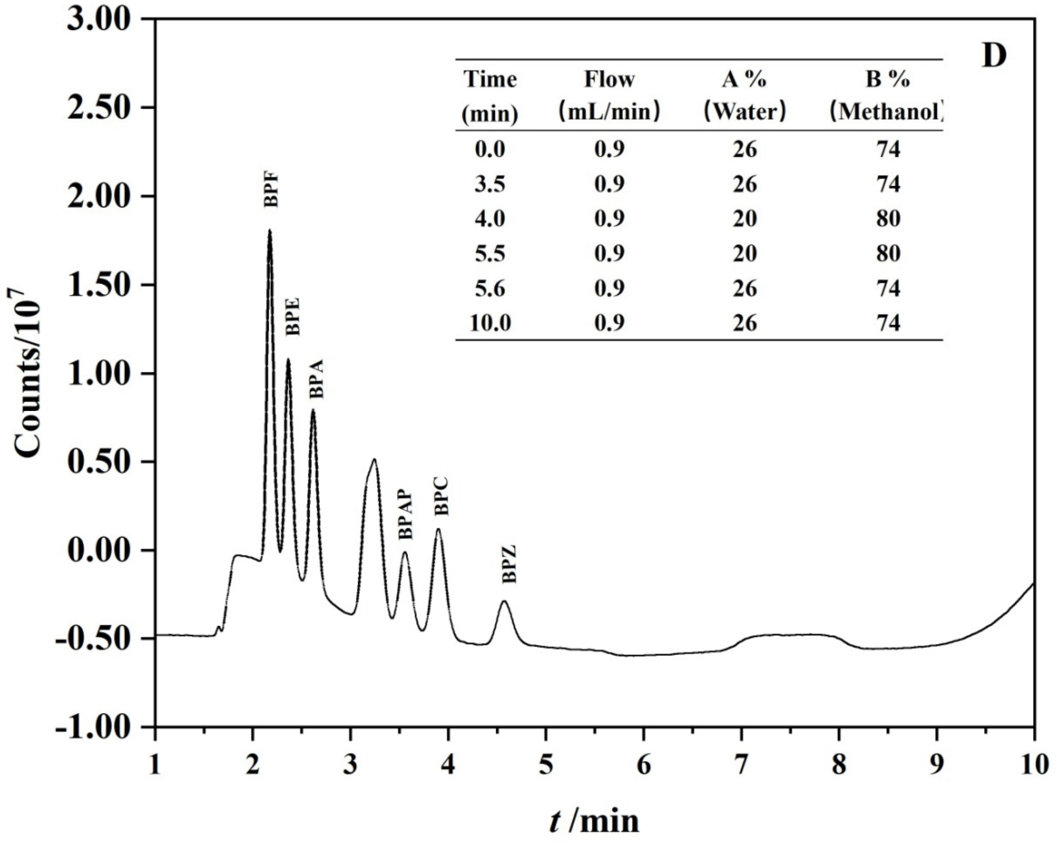
D
Time (min)
Flow (mL/min)
A % (Water)
B % (Methanol
0.0 0.9 26 74
3.5 0.9 26 74
4.0 0.9 20 80
5.5 0.9 20 80
5.6 0.9 26 74
10.0 0.9 26 74
BPF
BPE
BPA
BPAP
BPC
BPZ
Counts/10^7
t /min

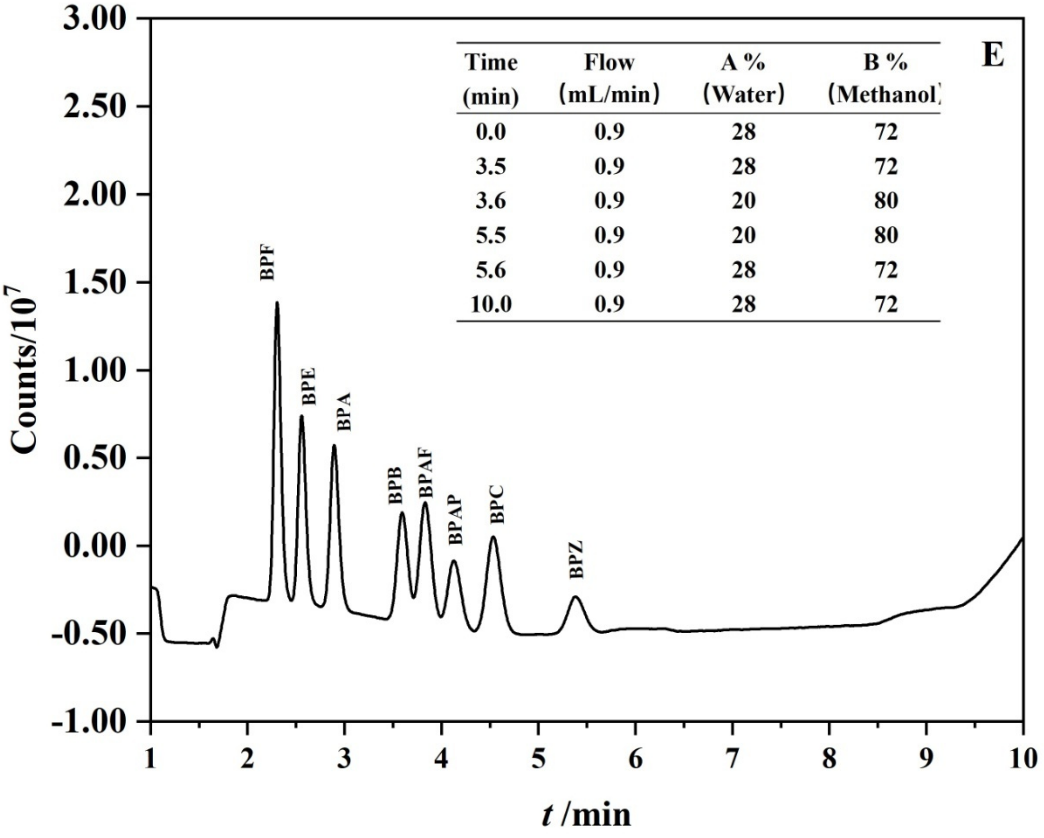

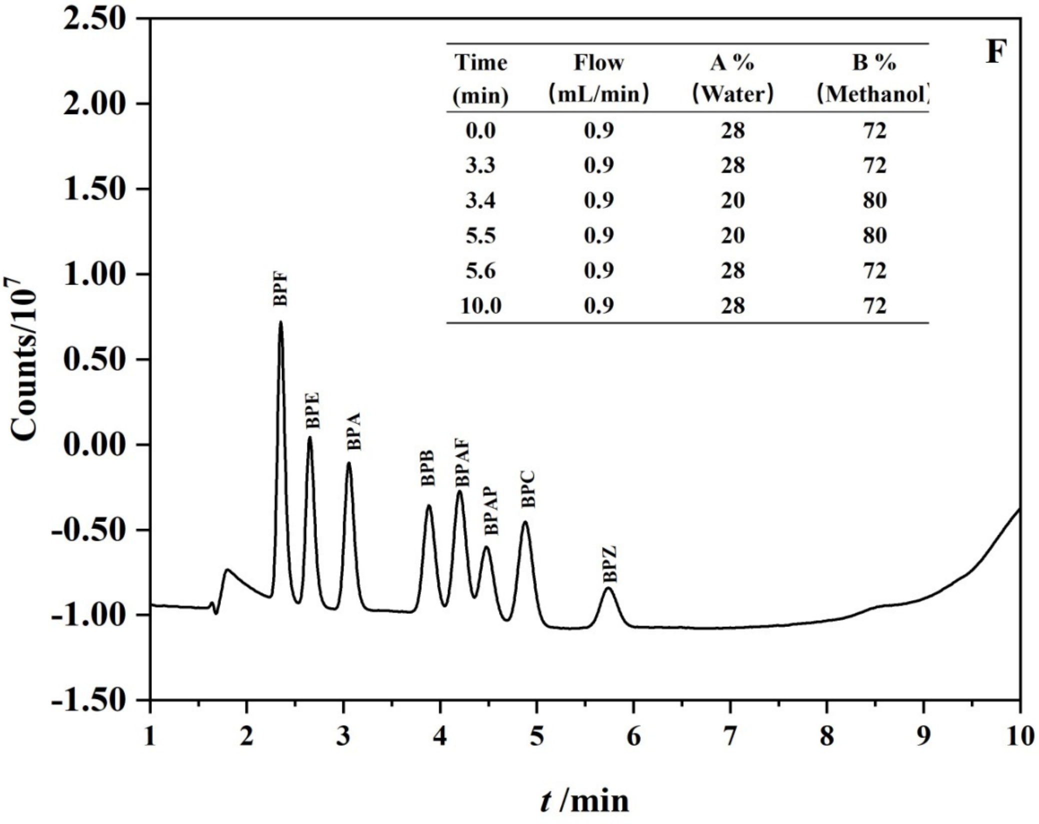

Figure S3. BPs separation under different gradient programs

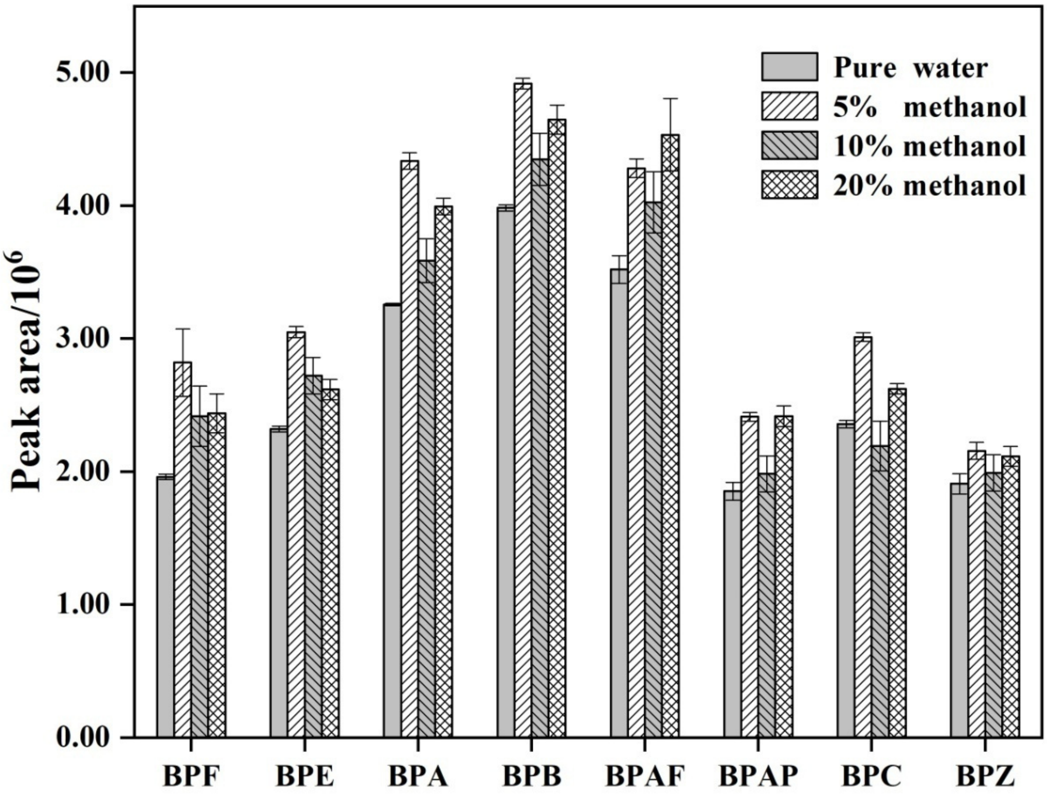

Figure S4. Comparison of peak area of the BPs at different carrier mobile phase

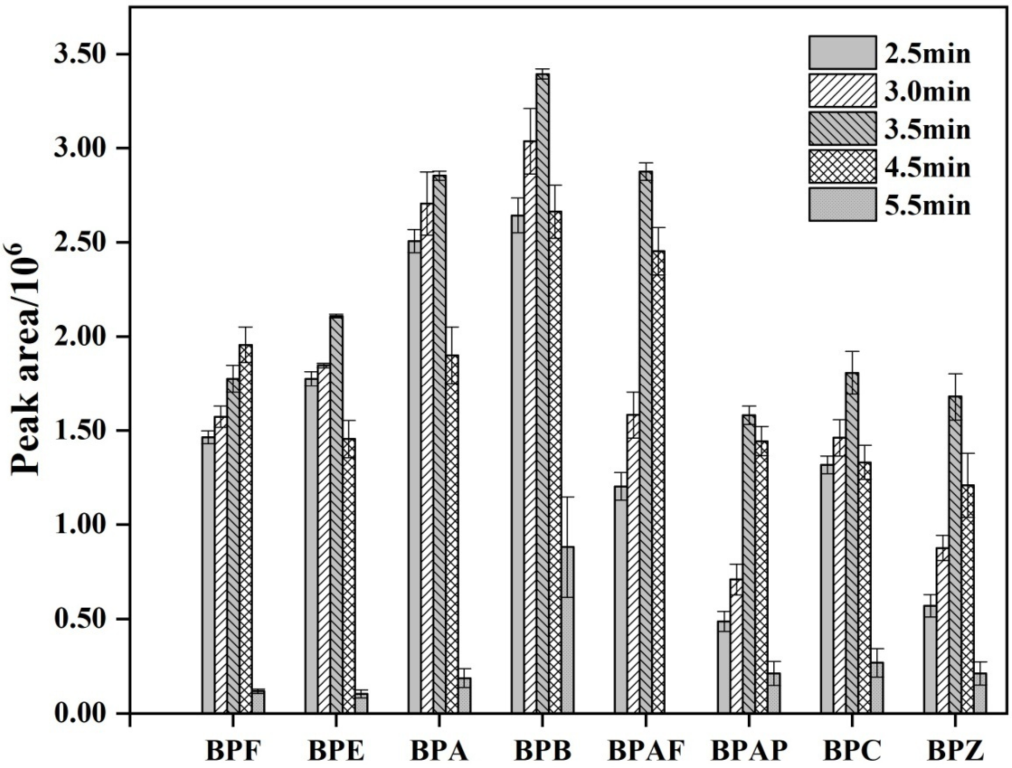

Figure S5. Comparison of peak area of the BPs at different durations

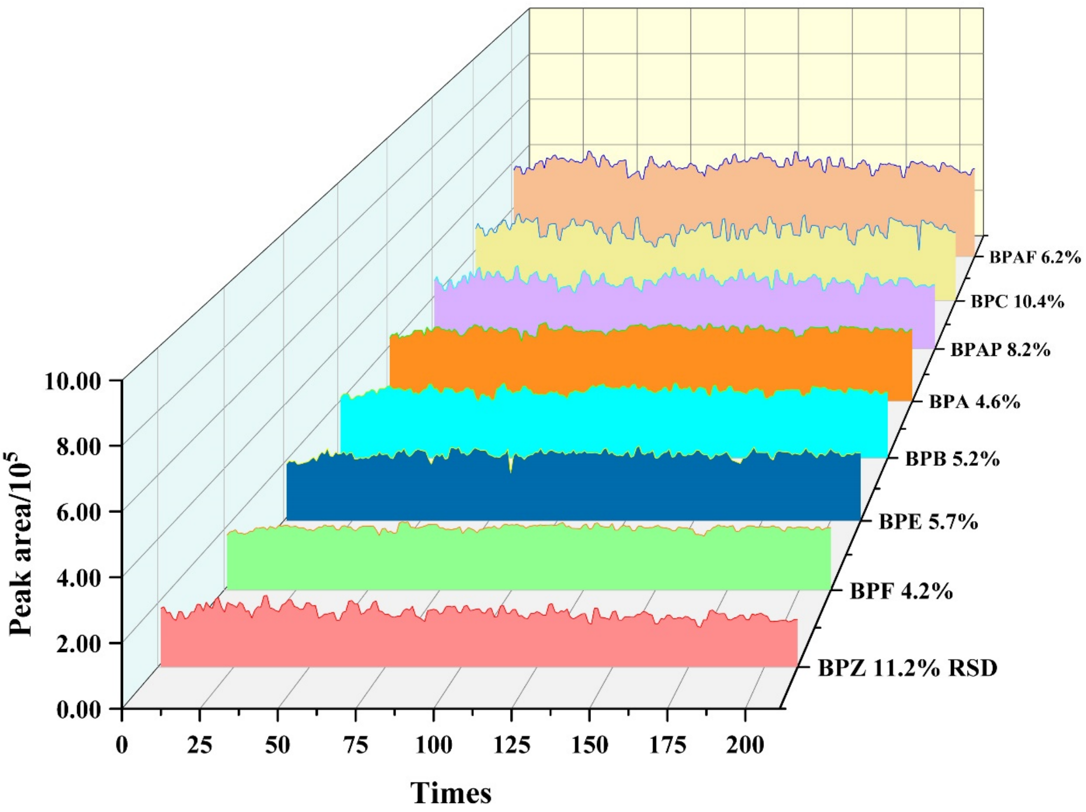

Figure S6. Change of peak area of BPs with the number of PFSPE columns used (10×2.1 mm PFSPE column, 5 ng/mL standard solution spiked in water)

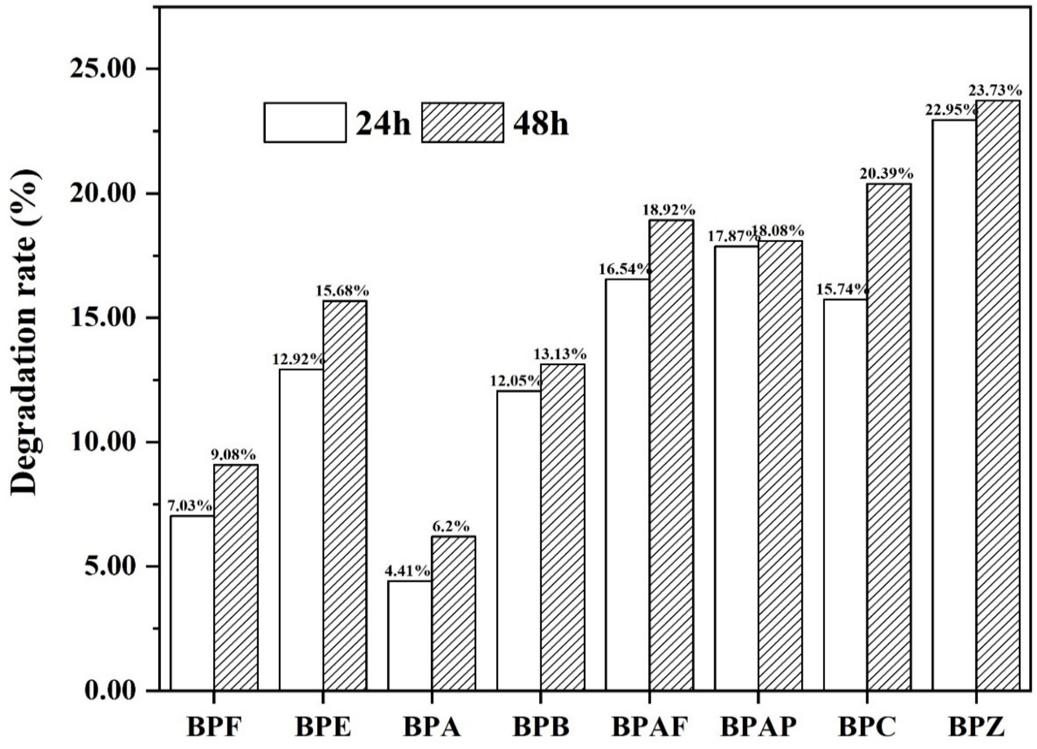

Figure S7. Degradation rate of 8 BPs at 24 h and 48 h (10×2.1 mm PFSPE column, 5 ng/mL standard solution spiked in water)

Table S1. The characteristics of drinking water samples

Sample No.	Ca^{2+} （mg/L）	Mg^{2+} （mg/L）	Na^{+} （mg/L）	K^{+} （mg/L）	H_2SiO_3 （mg/L ）	Total dissolved solids (mg/L)	pH
01	-	0.5-10	-	3-35	-	-	-
02	≥4.0	≥0.5	≥0.8	≥0.35	≥1.8	-	6.8-7.8
02-4	4.0-20.0	0.5-10	0.8-20.0	0.35-7.0	1.8-50	20-100	-
03	10-27	5-18	5-18	1-8	30-50	100-320	7.2-8.5
04-06	-	-	-	-	-	-	-
07	3.5-8.5	1.5-8.0	1.5-5.5	0.8-3.5	25-45	50-160	7-8
08	-	-	-	-	-	-	-
09	4-10	1.5-5	2-6.8	1-2.5	30-50	35-100	-
10	15-30	2-5	2-10	0.5-2	15-45	80-200	7.1-8.5
11	2-15	1-8	1-8	0.5-3	30-70	50-180	7-8
12	4-17	0.1-10	1-15	0.5-10	25-70	50-180	-
13	80	26	6.5	1	-	345	7.2
14	-	-	-	-	-	-	-
15	20-67.2	1.4-6.6	0.4-1.6	0.1-1	-	93-291.6	-
16	2-10	2-9	5-15	1-6	>50	80-200	-
17	7-20	6-20	3-16	2-8	30-50	70-300	-
18	1-30.8	1.0-7.2	2.6-18.8	1.0-6.8	30-50	40-160	-
19	35-65	3.5-10	-	0.2-3	20-80	80-400	7.2-8.3

-: there is no specific information

Table S2. The details and physical properties of drinking water samples

Sample No.	bottle color	material	date			Volume (L)
			Sampling date	Production Date	expiration date	
01	transparent	PET	2021.12.30	2021.05.09	2022.05.09	0.550
02-1	transparent	PET	2021.12.30	2019.09.29	2021.09.29	0.550
02-2	transparent	PET	2021.12.30	2021.08.19	2023.08.19	0.550
02-3	transparent	PET	2021.12.30	2021.04.15	2023.04.15	0.380
02-4	transparent	PET	2021.12.31	2021.11.05	2023.05.05	1.000
03	transparent	PET	2021.12.30	2021.04.29	2022.10.29	0.330
04-1	transparent	PET	2021.12.30	2021.09.13	2023.03.13	0.550
04-2	transparent	PET	2021.12.30	2021.09.25	2023.03.25	0.550
05	light blue	PET	2021.12.30	2021.11.30	2022.11.30	0.550
06	light blue	PET	2021.12.30	2021.10.22	2022.10.22	0.560
07	transparent	PET	2021.12.30	2021.08.01	2022.02.01	0.520
08-1	transparent	PET	2021.12.30	2021.09.24	2022.09.24	0.555
08-2	transparent	PET	2021.12.30	2021.03.30	2021.03.30	0.555
09	transparent	PET	2021.12.30	2021.05.25	2023.05.25	0.535
10	transparent	PET	2021.12.30	2020.08.04	2022.08.04	0.350
11	transparent	PET	2021.12.30	2021.04.08	2022.10.08	0.430
12	light blue	PET	2021.12.30	2021.07.10	2022.07.10	0.348
13	light blue	PET	2021.12.30	2021.01.05	2023.01.05	0.330
14	light blue	PET	2021.12.30	2021.03.06	2022.03.06	0.550
15	transparent	PET	2021.12.30	2021.03.21	2023.03.21	0.330
16	transparent	PET	2021.12.30	2021.04.15	2022.10.15	0.570
17	transparent	PET	2021.12.30	2021.07.02	2023.01.02	0.550
18	transparent	PET	2021.12.31	2021.04.22	2023.04.22	0.333
19	transparent	PET	2021.01.04	2021.11.12	2023.11.12	0.350
20	light blue	-	2021.12.30	2021.11.29	2022.03.01	-
21	dark blue	-	2021.12.30	-	-	-
22	dark blue	-	2021.12.30	-	-	-
23-28	-	-	2021.01.04	-	-	-

-: there is no specific information
